# Preparation of students with disabilities to graduate into professions in the South African context of higher learning: Obstacles and opportunities

**DOI:** 10.4102/ajod.v5i1.150

**Published:** 2016-02-24

**Authors:** Sibonokuhle Ndlovu, Elizabeth Walton

**Affiliations:** 1 Wits School of Education, University of Witwatersrand, South Africa; 2 Gender, Race and Identity Studies, University of KwaZulu-Natal, South Africa

## Abstract

**Background:**

Persons with disabilities continue to be excluded from professions in South Africa despite legislation on non-discrimination and equity.

**Objectives:**

We sought to identify both the opportunities and obstacles that students with disabilities face in professional degrees.

**Method:**

Selected texts from the South African and international literature were analysed and synthesised.

**Results:**

Students with disabilities are afforded opportunities to graduate into professions through the current climate of transformation, inclusion and disability policies, various support structures and funding. These opportunities are mitigated by obstacles at both the higher education site and at the workplace. At university, they may experience difficulties in accessing the curriculum, disability units may be limited in the support they can offer, policies may not be implemented, funding is found to be inadequate and the built environment may be inaccessible. Fieldwork poses additional obstacles in terms of public transport which is not accessible to students with disabilities; a lack of higher education support extended to the field sites, and buildings not designed for access by people with disabilities. At both sites, students are impacted by negative attitudes and continued assumptions that disability results from individual deficit, rather than exclusionary practices and pressures.

**Conclusion:**

It is in the uniqueness of professional preparation, with its high demands of both theory and practice that poses particular obstacles for students with disabilities. We argue for the development of self-advocacy for students with disabilities, ongoing institutional and societal transformation and further research into the experiences of students with disabilities studying for professional degrees.

## Introduction confirm

South Africa has legislation and policies that protect the rights of people with disabilities and promote their advancement (Republic of South Africa 1996, 1998, 2000). The reality on the ground, however, is that people with disabilities continue to be excluded from professional work. Disability is defined as difficulties confronted in functioning because of impairment or activity limitations (Statistics South Africa 2012), and using this definition, persons with disabilities are revealed to make up 10% of the total population. The Commission for employment equity, which was carried out in the years 2009–2010, indicated that 3909 persons with disabilities were professionally qualified and employed, which translates to about 0.6% of the total disability population (Ramutloa 2010). The figures suggest that very few persons with disabilities acquire professional degrees, and even those who do are excluded from professional jobs. This exclusion might result from non-implementation of equity and non-discrimination policies (Maja *et al.* 2011). Employers could also hold the view that persons with disabilities are inadequately skilled for the professional labour market (Swartz & Schneider 2006). Addressing this issue is important because persons with disabilities should be seen actively participating in the skilled labour force that South Africa seeks for the 21st century (Carrim & Wangenge-Ouma 2012). As the supply of professional skills is dependent on the output from Higher Education Institutions (HEIs) (Earlie 2008), it is necessary to consider what obstacles and opportunities students with disabilities encounter in HEIs as they prepare to enter the professional workplace.

This article critically explores the current literature relevant to the professional preparation of students with disabilities. We reviewed the South African literature from selected books, journal articles and the internet in order to understand the obstacles confronting students with disabilities as well as the opportunities available to them. We scanned the literature available on Google Scholar and ProQuest, as well as dissertations on the University of Witwatersrand’s Library Catalogue, using a combination of the search terms ‘disability’, ‘professions’, ‘students with disabilities’, ‘access’, ‘transformation’ and ‘inclusion’. This search yielded 65 texts, published between 1970 and 2015. These texts included South African and international books, book chapters, peer reviewed journal articles, policy documents, the Constitution of South Africa, research reports, as well as online resources like unpublished conference and discussion papers. The international literature has also been included in this article for a broad comparison of the higher education of students with disabilities. We set the scene by discussing professions and their preparation. This is important because not only are professional degrees different from other degrees, the very nature of what counts as ‘professions’ is contentious (Turner & Hodge 1970). The main claim of this article rests on the unique characteristics of professions, which make them particularly difficult to access by students with disabilities. After describing the opportunities and obstacles to the professionalisation of students with disabilities, we suggest, based on research, that self-advocacy could enable students with disabilities to confront some of the obstacles they face. We conclude by arguing for institutional reform through identifying and addressing exclusionary practices and call for further research into the experiences of students with disabilities studying professional degrees.

## The nature of professions and professional degrees

The nature of professional knowledge makes professional degrees different from other degrees in higher learning. A professional degree is a ‘high level qualification’ (Macdonald 1995:161) characterised by the accumulation of esoteric or abstract knowledge which can be applied in complex situations (Abbott 1988). Professional expertise operates as professionals draw on theoretical knowledge to inform their judgments and action in practice (Winch 2014). This requires, says Freidson (2001:35), ‘a foundation in abstract concepts and formal learning’. Professional curricula, thus, have unique characteristics in that they require both theory and practical application. Shay (2013:575) distinguishes professional curricula from theoretical curricula by saying that the logic of professional curricula ‘is the demands of the practice’. She also explains that professional curricula differ from practical curricula in that ‘the principles informing the practice are derived from theory’. Students graduating into professions, thus, need knowledge of theories and the ability to recognise the contexts to which this theory applies (Clarke & Winch 2004). The development of professional reasoning, judgment and action is a vital part of professional education and, says Winch (2014:58), should be ‘reflected in appropriate assessment arrangements linked to professional curricula’. This article argues that it is in the uniqueness of professional preparation, with its high demands on both theory and practical application, that poses particular obstacles for students with disabilities.

Professional degrees are distinct from other programmes in that they are associated with professional bodies who accredit them (Harvey & Mason 1995). Accreditation is based on the suitability of the institution to offer a particular professional degree. For example, during apartheid, the professional degree of architecture was only offered in internationally recognised White institutions (Van Rensburg 2011), meaning that historically disadvantaged institutions could not offer architecture because they were not accredited to do so. Professional bodies are also involved in the design of the curriculum of the respective professional degrees in higher learning (Jamal & Bowie 1995). According to Harvey & Mason, ‘Professional bodies define the specific competencies, including the underpinning knowledge, that are required by graduates for them to be effective practitioners’ (Harvey & Mason 1995:1). Professional bodies are not only involved in curriculum design, defining competencies and prescribing specific knowledge, but are further involved in assessing competence in practice. Though the procedure might not be standard for all professional degrees, students in these programmes are examined for their grasp of academic theory in higher learning and are further examined for competence in practice by the relevant professional bodies.

Professionalisation is a term used to refer to learning for a professional occupation. Whilst the term has been used differently in various contexts, we use the term in line with Griffin, Green and Medhurst’s (2005) view that it is the way in which people are acculturated into an academic discipline and shaped, so as to be recognised, legitimised and accommodated as professionals in the working context in which they will operate. In higher learning the academic staff play a very important role in the professionalisation of students with and without disabilities (Vickerman & Blundell 2010). Lecturers themselves are regarded as professionals because lecturing in itself is categorised as a higher level profession (Haralambos & Holborn 1991).

The concept of professions has developed over time. In the past, professions have been regarded as having power in themselves (Barber 1963, cited in Haralambos & Holborn 1991) because of the specialised knowledge, which was only accessible to those within the profession. Delivery of unique professional services was, therefore, ‘highly regarded in terms of society’s values’ (Baber 1963 cited in Haralambos & Holborn 1991:67). Thus, professions were not only seen as different from other occupations but were also seen as yielding power and status in society. Challenging this, Taylor and Runté (1995) have questioned the very notion of a profession, arguing that those who cling to the idea of professions do not want to surrender their superiority. We do not agree that the idea of professions is obsolete, but do note that in some professional curricula there has been a weakening of traditional and specialised disciplinary knowledge as other related disciplinary knowledge has been integrated into programmes (for example, as business courses are introduced into medical degrees). As a result, it could be argued that professions are no longer as distinctive and autonomous as they used to be.

Professions can be viewed as different from other occupations because they are informed and guided by moral values and ethics (Higgs-Kleyn & Kapelianis 1999). This resonates with Winch’s (2014:58) view that the judgments made by professionals are ‘not usually just technical ones but also involve ethical and political considerations to which one’s personal and occupational values are highly relevant’. Harvey and Mason (1995) argue that the professional bodies are responsible for monitoring conduct and ensuring that members abide by the profession’s established ethical principles as a means for internal and external quality (Harvey & Mason 1995). Grace (2014) disagrees, believing that the ethical and moral fibre that makes professions different from other occupations is lost in the contemporary context because of capitalism and global economic marketisation. This argument sees that the world has shifted to the commodification of professions and moral and ethical principles have been replaced by monetary value. The existence of professions within such a context has made them similar to any occupation seeking monetary value. We concede that moral conduct and ethical principles may experience degradation, but argue that the moral and ethical principles upholding the uniqueness of professions still exist. In the South African context, professional bodies still gate-keep and monitor professions for conduct and abide by ethical principles. Registration with the respective professional bodies is a prerequisite for practice and we suggest that with professional bodies in control, professionals will continue to practise within a context of formalised ethical principles.

Professions clearly differ from one another. However, for the purpose of this article, the HEI preparation of students to graduate with professional degrees has been homogenised. This is because there are common requirements for preparation across a range of professions (Badza & Chakuchichi 2000; McEachern & Kenny 2007). HEI preparation ‘to graduate into professions’ refers to professionalising students to be ready to enter into a respective profession after obtaining a professional degree. Thus, we understand preparation to enter into professions as the whole process of acquisition of professional knowledge in higher learning, in order to apply it with professional expertise and professional judgement in practical contexts (most often the workplace). We now move to discuss the main obstacles and opportunities in preparation to graduate into a profession that is highlighted in the literature, having established the distinctiveness of professions and professional degrees.

## Opportunities and obstacles in professional preparation

Opportunities and obstacles that students with disabilities might be confronted with, during their preparation to graduate into professions, could be similar to those that students without disabilities face. This is because preparation is standardised for all students and all students must meet the same requirements for graduation and professional registration. We show in this section that there could be additional obstacles and opportunities that are specific to students with disabilities because of their unique needs.

### Opportunities

The opportunities described below refer to structural, policy and material support that should be available to students with disabilities, and should enhance their access to and success in professional degrees.

## Climate of transformation

The post-apartheid climate of institutional transformation is a potential opportunity for students with disabilities. During apartheid, the South African schooling system was segregated according to race and disability (Howell 2006). In higher learning segregation was only implemented along racial lines (Howell 2005), which means that students with disabilities have never been explicitly excluded from HEIs. However, as Howell, Chalklen and Alberts (2006) note, with respect to persons with disabilities, ‘… attitudes and institutional practices … have perpetuated some of the deepest inequalities and most severe forms of discrimination in our country’s history’ (p. 78). Students with disabilities have experienced discrimination and exclusion effected by institutional practices that work to the benefit of students without disabilities. With disability now firmly placed on the transformation agenda (Howell *et al*. 2006), students with disabilities are now represented on the transformation committees that South African HEIs have formed (DoE 2008). The increased awareness of the rights of persons with disabilities, buttressed by the policies described below, should offer improved opportunities for students with disabilities to pursue professional degrees.

## Inclusion and disability policies

South Africa has comprehensive and specific policies for inclusivity in education and training for employment (Department of Education [DoE] 2001a; Department of Higher Education and Training 2013) which indicate political support for the education of learners and students with disabilities. The policies cover general, further and higher education and are concerned with addressing barriers to learning, promoting institutional access for students with disabilities, as well as planning for, and providing appropriate support up to the point of employment. These policies should enable the professionalisation of students with disabilities on two levels. Firstly, the level of schooling is involved, where improved access to quality education for learners with disabilities is envisaged. This should result in improved schooling outcomes for learners with disabilities, and ultimately in their meeting the admission requirements for entry into professional degrees. Secondly, the provisions of the Education White Paper Six (DoE 2001a) make reference to HEIs improving access and support at institutional level. The provisions of the White Paper for Post-School Education (DHET 2013) specifically refer to HEIs providing training for people with disabilities to prepare them for the labour market. The inclusion of students with disabilities in higher learning is also backed by institutional disability policies. There has been an increase in institutions that have disability policies (Fotim Report 2011) and 21% of institutions surveyed by Matshedisho (2007) were using formal policies to provide support for people with disabilities. The rhetoric of policy does not necessarily translate into practice. Inclusion policies are known for their contradictory discourses (Liasidou 2012), particularly as they simultaneously espouse the individual or deficit and social accounts of disability. These contradictions may account for the ‘gap’ between policy and practice (Pather 2011), and may explain why policy does not always translate into opportunities for students with disabilities.

## Support for students with disabilities

There is also the opportunity of a high level of disability support. Disability Unit staff play an important role in providing direct and indirect support to students. Direct support is usually technical and material through the provision of assistive devices, services and assistance with administrative procedures. Indirect support occurs as Disability Unit staff train lecturers, and work collaboratively with them in teaching students with different categories of disabilities (Matshedisho 2007). According to the United Nations Convention on the Rights of Persons with Disabilities (United Nations 2006) to which South Africa is a signatory, lecturers are obliged to make ‘reasonable accommodations’ for students with disabilities. Whilst there might be contestations about what constitutes ‘reasonable’, it would be expected that access arrangements would be made for assessments, that there would be adjustments to the delivery of courses, and the provision of course material in an accessible format (Marshall 2008). This, of course, depends on the lecturers’ willingness to teach in ways that include students with disabilities. The opportunity of a high level of professionalisation for students with disabilities could be achieved through the coordinated support of the academics and Disability Unit staff in South African HEIs.

## Funding

Funding is important for all students if they are to be successfully prepared in higher learning and to graduate into professions. In 1996, South Africa introduced the National Student Financial Aid Scheme (NSFAS 2013) to fund needy but capable students in higher learning (Carrim & Wangenge-Ouma 2012). Before 2008, students with disabilities had their own funding provided by the Department of Labour under the National Skills Fund (NSFAS 2013), to assist them in studying professional and non-professional degrees. In 2008, the Department of Education introduced a special NSFAS bursary specifically for students with disabilities (NSFAS 2013). The bursary covers students with disabilities who were previously funded through the Department of Labour, and is for undergraduates studying any degree and post-graduates studying professional degrees (NSFAS 2013). The NSFAS bursary for students with disabilities covers the students’ tuition, accommodation, meals, transport costs, costs of material prescribed by the institution and the cost of one or more assistive devices (NSFAS 2013). A particular opportunity is presented by the provision of funds to cover transport expenses. As fieldwork practice, for professional degrees in the South African context, is off campus in most instances (Odendaal-Magwaza & Farman 1997), all students studying professional degrees have transport costs. Students with disabilities enjoy the opportunity of financial support that is specifically for transportation to the field, during field practice. From the list of expenses for which the NSFAS bursary makes provision, it seems that the students with disabilities should be fully financially covered to be successfully prepared to graduate into professions in South Africa.

### Obstacles

We have, in describing various opportunities, hinted at the fact that these may not be sufficient in the quest for the professionalisation of students with disabilities. In the sections that follow, we discuss obstacles that students with disabilities confront first at the HEI site and then in fieldwork. In so doing, we build the argument that it is in the nature of professional (as opposed to purely theoretical or purely practical) education that the obstacles for students with disabilities are compounded.

## Obstacles at the university site

Students with disabilities may confront obstacles to accessing professional curricula in formerly advantaged institutions in South Africa. Research on transformation in these contexts has revealed that some lecturers are not willing to make changes to the curriculum to enable access for formerly disadvantaged social groups (DoE 2008). This may be exacerbated by the association of disability with incapability in the South Africa context of higher learning (Howell 2006). We could extrapolate conclusions from this and assume that negative perceptions of the capabilities of students, with disabilities and low expectations of their academic performance, could be held by academic staff who are responsible for these students’ professionalisation. Professional degrees are academically demanding (Haralambos & Holborn 1991) and students might choose not to disclose invisible disabilities for fear of being labelled as incapable (Fuller *et al.* 2004; Goode 2007). As a consequence, students with disabilities might not receive the support and accommodation to which they are entitled and this may impact on their ability to acquire the theoretical knowledge that is required for professional expertise.

Whilst support from Disability Units potentially enables the access and success of students with disabilities, the Fotim Report (2011) notes that Disability Units have minimal autonomy and direct communication with university management. This constrains their support for students with disabilities and indirectly leads to academic staff having a lower level of participation, negotiation and awareness regarding disability issues (Lyner-Cleophas *et al*. 2014). The White Paper for Post-School Education and Training (DHET 2013) proposed a coordinated approach that includes support from the support staff, academic staff and management. The view of Lyner-Cleophas *et al*. (2014) coordinated support as a systemic approach that could make a positive impact on the inclusion of students with disabilities in higher learning and this could improve the professionalisation of students with disabilities. However, as we explain next, policy ideals are not always realised in practice.

Despite comprehensive policies of inclusive education, inclusion in higher learning in South Africa is problematic (Carrim 2002). The exclusion of students from disadvantaged backgrounds in general and students with disabilities in particular is still being experienced in the South African context of higher learning (DoE 2008). The specific policies regarding disability in higher learning in South Africa are not effectively implemented and in many instances, disability policies have taken a long time to merely be approved by management structures (Fotim Report 2011). Institutions of higher learning in South Africa also do not have a specific way of monitoring the implementation of disability policies as is found in more developed countries (Chataika 2007). Preparation to graduate into professions might be backed by policy, but when policy is not translated into action, professionalisation in higher learning could be a far-fetched dream for students with disabilities.

Despite the apparent opportunity for funding students with disabilities, the reality is that the ‘NSFAS is currently the only state funding body in South Africa and, therefore, very few students with disabilities are able to access higher education and succeed in their studies’ (Fotim Report 2011:137). Further research is required to understand why the funding provisions envisaged by the NSFAS are not resulting in access and success in higher education for students with disabilities. Mention needs to be made of the fact that by virtue of being expensive, professional degrees exclude students of low socio-economic status (Le Grange 2014). These degrees require more funding because of costs like clinical supervision and specialised and expensive equipment. The degree programme of architecture, for example, is the most expensive programme in higher learning, with fewer students from disadvantaged backgrounds (like Black Africans and people with disabilities) entering and completing this degree (Le Grange 2014). Although there is NSFAS funding specifically for students with disabilities, it might not be adequate for studying professional degrees in higher learning.

None of the South African universities were originally built with the needs of students with disabilities in mind (Fitchett 2015). As a result, students with disabilities who are studying professional degrees confront obstacles in accessing lecture venues (Hall & Belch 2000; Losinsky *et al.* 2003). Where rails and ramps are available, they are usually at the back of buildings. As a result, students with disabilities are obliged to spend extra time getting to venues and could miss lectures altogether, which could affect their academic performance. Although retrofitting is being implemented in some institutions of higher learning (Fitchett 2015) this is a long-term endeavour. The inaccessible environment in South African higher learning has implications beyond access to buildings. Fitchett (2015) reports that a particular South African HEI has started to build new structures with access for people with disabilities in mind. Despite this, students with disabilities report that the new buildings are still problematic because there is too big a space between the sitting areas, the podium and the board. This suggests that the construction has not complied with specifications on spaces and sizes in Principle 7 of Universal Design. This states that there should be appropriate size and space for use by all users despite body size, posture and mobility (Centre for Universal Design 1997). In those big spaces, students with low vision might not see what is written on the board from where they are sitting. Students with hearing impairments might not hear clearly when the lecturers teach from the podium. Students who use wheelchairs are disadvantaged when tables and chairs require access from stairs. These built environment obstacles have negative implications for the professional preparation of students with disabilities, particularly as they potentially limit the students’ access to the theoretical knowledge taught at the HEI site.

## Obstacles to practical and fieldwork experience

Preparation to graduate into professions involves practice and experience in the field. As has been mentioned, fieldwork practice for professional degrees mostly takes place off campus at workplaces (Odendaal-Magwaza & Farman 1997). This, then, poses obstacles for students with disabilities over and above those experienced at the HEI site. Students with disabilities may need the support available to them at the HEI site extended to include support in fieldwork. In the British context, there is extended support by higher learning into the field (Botham & Nicholson 2014) but we can find no evidence that this occurs in the South African context. Without extended support, students with disabilities might experience difficulties during fieldwork and this may impact negatively on their professionalisation.

The first of the fieldwork obstacles is transport. Most public transport in South Africa remains inaccessible to persons with disabilities, especially those using wheelchairs (Khuzwayo 2011). The few public transport facilities that are accessible are available in urban areas (Parliamentary Monitoring Group 2013). Additionally, fieldwork for professional degrees is not limited to urban areas. Students using wheelchairs often find that there is a lack of space for their wheelchairs in public transport. Also, the ‘normal’ entrance of the vehicle and the distance from the ground to the entrance of the vehicle is problematic (Khuzwayo 2011). Inaccessible transport to fieldwork sites, thus, has the potential of exerting a negative effect on the professionalisation of students with disabilities. Once in the field, students with disabilities may find built environments that constitute further obstacles to them preparing for their professions (Losinsky *et al.* 2003). Many South African workplace environments were not originally designed with the needs of persons with disabilities in mind and Swartz and Schneider (2006:235) argue that ‘retro-fitting existing buildings and access routes to accommodate all South Africans can be technically and aesthetically challenging, not to mention expensive’. Where retro-fitting does occur, says Fitchett (2015), building owners usually meet only the minimum requirements in compliance with the National Building Regulation of South Africa.

## Negative attitudes and assumptions of individual deficit

Students with disabilities are confronted with the obstacle of the reproduction of negative attitudes towards them (Howell 2006). This reproduction of negative attitudes in higher learning and in the field emanates from people viewing disability in a negative light. Watermeyer and Swartz (2006) talk of the ‘hostile and patronising attitudes’ (p.1) that people with disabilities in South Africa experience. Many identity markers may lead to negative attitudes by others, but Howell (2006) found that negative attitudes towards students with disabilities in South African higher learning were more pronounced, especially for those from low socio-economic backgrounds, who are sometimes referred to as ‘non-traditional’ students (DoE 2001b). As negative attitudes take a long time to change, students with disabilities continue to experience negative attitudes that hinder full preparation to graduate into professions. South Africa is not alone in this. Other countries also report attitudinal barriers limiting the optimal functioning of students with disabilities in higher learning (Chataika 2007; Holloway 2001).

Negative attitudes combine with continued individual and deficit understandings of disability to create obstacles for students with disabilities. Despite some shift in the South African HEIs from understanding disability within an individual model to understanding it within a social model, the individual or deficit understanding prevails (Fotim Report 2011). The individual model perpetuates the idea that disability is an individual problem requiring individual compensatory measures (Oliver 1996). This approach sees disability as inherent in the individual, rather than socially constructed by a disabling society. The model prevents disability from being seen as oppression and is focussed on enabling functionality for individuals, rather than identifying and dismantling barriers to full access and participation. Disabilities in students might be considered individual tragedies and as a result, the service provided may be seen as charity, rather than the right of students with disabilities. Taken together, these negative attitudes may explain the lack of full participation of students with disabilities both in higher learning and in fieldwork. We consider perpetuation of the individual model as an obstacle that could have negative implications for the preparation of students with disabilities to graduate into professions.

We have, for ease of explanation, considered the various opportunities and obstacles experienced by students with disabilities under discrete headings. This belies the compounding effect of the combined obstacles. The fact that buildings in HEIs and workplaces, as well as transport, remain inaccessible suggests that society continues to believe that it is the person with a disability who is responsible for arranging access to the physical environment. It is, thus, important that society becomes conscious of the barriers for persons with disabilities that are encountered in the physical environment (Oliver 1996; Oliver & Barnes 2012). This means ensuring that physical access for people with disabilities is seen as a social issue, and not a personal problem (Slee 2011). This is particularly relevant in South Africa, where there is a general belief that the presence of persons with disabilities in the workplace might mean incurring extra costs to make the environment disability-friendly (Marescia 2003). Besides confronting the obstacles of inaccessible transport and buildings, students with disabilities are confronted with the obstacle of social discrimination (Hall & Belch 2000) during fieldwork and in workplaces (Marescia 2003). Also, their potential and capabilities may not be recognised by staff in the field (Wiggert-Barnard & Swartz 2012).

## Self-advocacy as a way to overcome the obstacles

Self-advocacy may be a way that students with disabilities could challenge the obstacles in higher learning and in the workplace during field practice. In the South African context, Swart and Greyling (2011) reported on a study in which students with disabilities argued that self-advocacy was the way through which they could communicate their needs and demand support to which they are entitled. For students with disabilities to effectively self-advocate in higher learning and the workplace, they need to develop personal characteristics (Swart & Greyling 2011) and specific skills (Getzel & Thoma 2008). Personal characteristics like patience, friendliness, determination and agency are identified as important attributes for students with disabilities who wish to self-advocate, as they will need to negotiate and sometimes demand support (Swart & Greyling 2011). In addition, they need to know who they are, to believe in themselves, to know what works for each of them and to know what services should be provided (Swart & Greyling 2011). The skills necessary for self-advocacy include communication, problem solving and conflict resolution skills (Getzel & Thoma 2008). Students with disabilities need not view themselves as passive subjects, waiting upon academic staff, support staff and personnel in the field to professionalise them. In developed countries such as the USA, self-advocacy has been found to lead to successful outcomes in terms of employment (Test *et al*. 2005). The implication is that through active engagement, transformation occurs.

## Conclusion

After two decades of democracy, higher learning in South Africa has made strides in providing opportunities for students studying professional degrees in general, and students with disabilities in particular. However, a number of obstacles are still experienced, specifically by students with disabilities, which result in a lack of professional skills amongst persons with disabilities in the South African context. These obstacles interact to negatively influence the professionalisation of students with disabilities. It can, therefore, be concluded that the low representation of persons with disabilities in South Africa in professions can, to an extent, be explained by the obstacles they face in their preparation for professions in higher learning. Addressing these obstacles is crucial. It is imperative that the individual approach to disability is deconstructed and that higher education engages with residual discriminatory and exclusionary discourses and practices. In this regard, Walton, Bowman and Osman (2015) note about South African HEIs that:

… support for students is often framed in terms of a compensatory discourse, based on the assumption of student disadvantage or deficit. The institution, in this discourse, is assumed to be normative, and its demands unproblematic. (p. 269)

Whilst there has been research on the experiences of students with disabilities in higher education, more focus is required on the specific experiences of students with disabilities who are preparing to graduate into professions. The voices of students with disabilities need to be heard in research that is designed for participation and transformation (Mertens 2012). For change to occur in higher learning in favour of students with disabilities, empowerment and agency are needed. Self-advocacy, as reported by Swart and Greyling’s (2011) study, could also make a difference. Finally, it is incumbent on the schooling system to become more inclusive of learners with disabilities and to ensure that their education will give them access to higher learning to prepare for, and graduate into professions.
